# Adult diffuse hepatic hemangiomatosis

**DOI:** 10.4322/acr.2021.401

**Published:** 2022-09-23

**Authors:** Neha Bhardwaj, Mayur Parkhi, Manish Kumar, Lileswar Kaman, Suvradeep Mitra

**Affiliations:** 1 Post Graduate Institute and Medical Education and Research (PGIMER), Department of Pathology, Chandigarh, India; 2 Post Graduate Institute and Medical Education and Research (PGIMER), Department of Histopathology, Chandigarh, India; 3 Post Graduate Institute and Medical Education and Research (PGIMER), Department of General Surgery, Chandigarh, India

**Keywords:** Liver, Vascular Neoplasms, Hemangioma, Cavernous, Pathology

## Abstract

Diffuse hepatic hemangiomatosis (DHH) is an uncommon vascular lesion, though hemangiomas are the commonest benign tumors of the liver. The etiology is largely unknown to date; however, its association with giant cavernous hemangiomas (GCH) has been reported in the literature. We present herein, the case of a 37-year-old hypothyroid woman with abdominal fullness for 2 months. The contrast-enhanced computed tomography revealed multiple well-encapsulated lesions involving the liver lobes and was diagnosed as giant cavernous hemangiomas. Most of them, except the deep-seated ones, were enucleated. Histopathological examination highlighted the presence of GCH with irregular margin, replacement of hepatic parenchyma, and presence of multiple micro-hemangiomas suggesting the possibility of DHH further substantiated by retrospective radiological assessment. No extrahepatic vascular lesion was noted, and the post-operative recovery and follow-up were uneventful. Adult DHH is an uncommon entity. The diagnosis of DHH and its distinction from GCH are important from the management and prognostic point of view as recurrence, extrahepatic manifestations, features of consumption coagulopathy, and death from the complications are not uncommon.

## INTRODUCTION

Hemangiomas are the most common benign liver tumors, usually encountered as incidentalomas at laparotomy or autopsy. The prevalence of hemangioma, a benign vascular tumor, varies from 5 to 20% in different studies.^1^^,^^2^ Histomorphologically, the hepatic hemangiomas consist of large cavernous-sized vascular spaces lined by a uniform monolayer of the endothelium, a characteristic of cavernous hemangioma.^1^^,^^3^ The hepatic cavernous hemangiomas (CH) sometimes adopt a clinically apparent large size known as ‘Giant cavernous hemangioma (GCH)’. The prefix “giant” is used over an arbitrary cut-off of 4 to 8 cm in various studies.^2^^-^5 GCH carries a higher risk of spontaneous rupture and sequestration of platelets, requiring surgical intervention.^2^

Diffuse hepatic hemangiomatosis (DHH) is characterized by innumerable hemangiomas invading and replacing the liver parenchyma. DHH can be limited to a hepatic lobe or involve the hepatic parenchyma diffusely.^1^^,^^3^ DHH is an unusual lesion with a pediatric preponderance. A PubMed search till May, 2022 along with the literature review by He et al.^6^ showed only 19 reported cases of adult DHH depicted in the English literature.^3^^,^^4^^,^^6^^-^22 Approximately half of the DHH cases can be seen in association with GCH.^2^

The etiopathogenesis of DHH is uncertain, although it has been diagnosed in association with hereditary disorders, such as skeletal hemangiomatosis and Osler-Weber-Rendu disease, and drugs like oral contraceptives (OC) and metoclopramide.^9^^,^^20^^,^^23^ The final diagnosis is histopathological, as imaging tests are not effective in accurately differentiating their differential diagnoses. The clinical case presented here serves as an example to corroborate this point. Furthermore, the diagnosis of DHH mandates a thorough radiological evaluation to rule out any associated extrahepatic vascular lesion(s).^2^^,^^5^ We hereby present an unusual case of isolated adult DHH associated with multiple GCHs, diagnosed primarily by histopathology and retrospectively confirmed by radiology.

## CASE REPORT

A 37-year-old woman presented with right upper quadrant fullness and shortness of breath for 2 months. Her medical history included the diagnosis of hypothyroidism on L- thyroxin (25 mcg/day), antidepressants usage over the last 8 years, and was submitted to an uneventful open appendicectomy (gangrenous appendicitis with peritonitis) one month before the most recent complaints. She denied alcoholism, diabetes, or hypertension. Her liver function tests were normal apart from mild elevation of serum alanine aminotransferase (ALT) (47 IU/L; Normal range: 0-40 IU/L). The levels of carcinoembryonic antigen (CEA), CA-19-9, α-fetoprotein (AFP) were normal. The serological workup for all hepatotropic viruses was negative.

**Figure 1:**
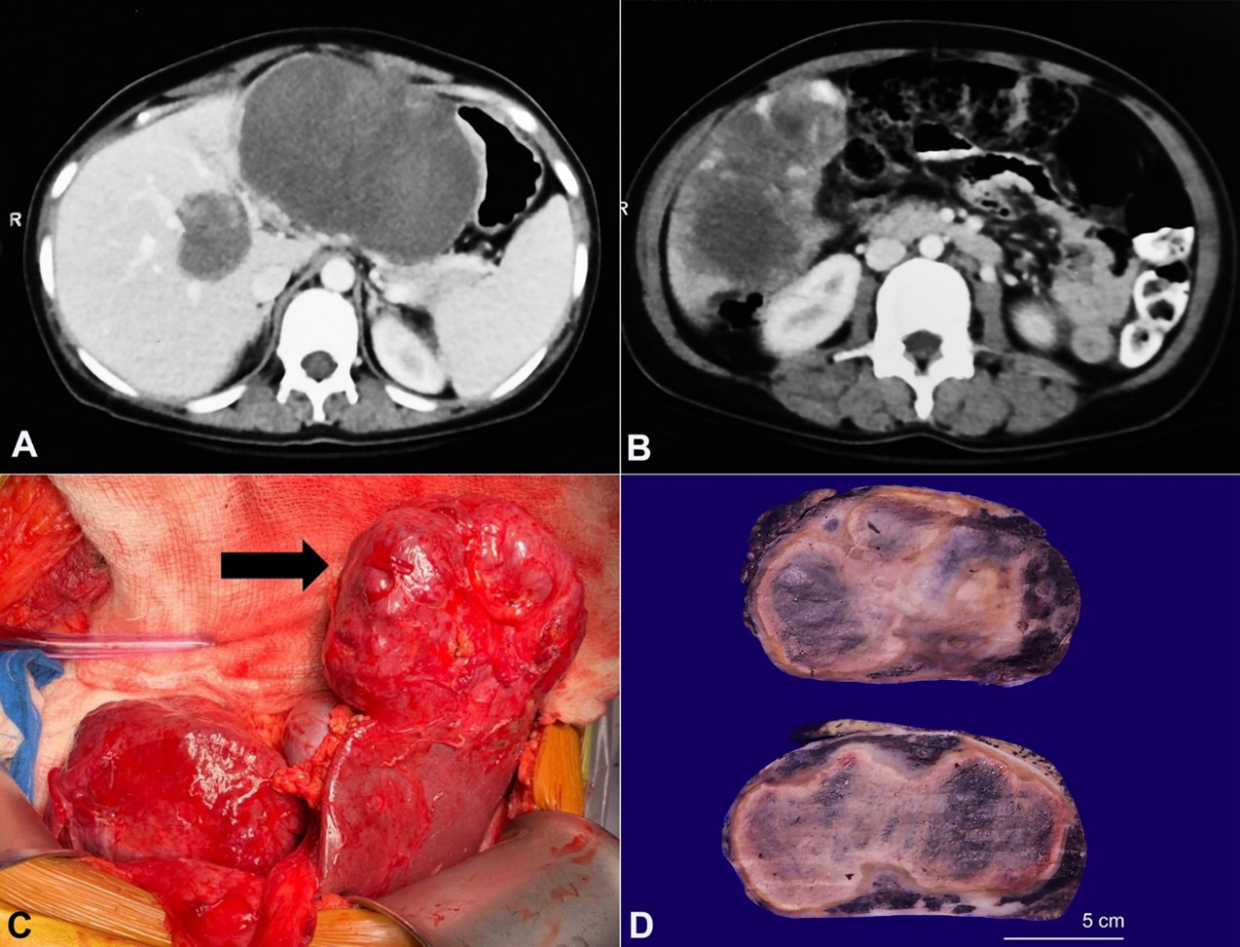
Radiology and gross images of the lesion(s). **A –** The CECT showing the largest hypodense lesion involving the left lobe along with its medial extension; **B –** The CECT showing a large heterogeneous and hypodense lesion in the right lobe with irregular margins; **C –** The intraoperative figure showing the large lesion in the left lobe of the liver (black arrow); **D –** The gross picture of the largest lesion highlighting a seemingly well-encapsulated mass with tan-brown color and a spongy honeycombed cut surface.

The contrast-enhanced computed tomography (CECT) scan showed an enlarged liver (liver span of 20 cm) with multiple heterogeneous hypodense lesions involving both the lobes. These lesions showed peripheral nodular enhancement. The largest lesion measuring 11×8×11.3 cm was located in the left lobe with medial extension in the gastrohepatic region abutting the gastric lesser curvature laterally, the antropyloric region inferiorly, and the body and the tail of the pancreas posteriorly (Figure 1A). Another large lesion sizing 10×7.5×8.2 cm was identified in the right hepatic lobe extending downwards to the pararenal space with irregular margins (Figure 1B). The hepatic and portal veins were patent. Thus, the diagnosis of multiple GCH was suggested. The imaging also revealed an enlarged spleen with normal attenuation; however, no focal lesion or other extrahepatic lesion was identified.

During surgical enucleation of the vascular masses, dense adhesions of the liver with both the anterior abdominal wall and transverse colon were noted (Figure 1C). The large lesions involving various anatomical segments were excised; A) a well-defined exophytic lesion (10×8 cm) involving segments VI and V abutting the gall bladder, B) the largest lesion (12×12 cm) completely replacing segments II and III, C) two smaller lesions (3×3 cm) involving segment IVb and segment VIII of the liver. A deep-seated lesion measuring 4×4 cm associated with the middle hepatic vein splaying was left *in situ* due to its proximity to these vessels.

The enucleation specimens from the right lobe, left lobe, and Segment VIII measured 8×6×4.5 cm, 9×7×5 cm, and 3.5×2×2 cm, respectively. The cut surfaces of all these specimens revealed well-encapsulated, dark red to tan-brown colored masses. These masses showed a firm to a spongy cut surface with a few perilesional cherry-colored honeycomb foci (Figure 1D).

**Figure 2:**
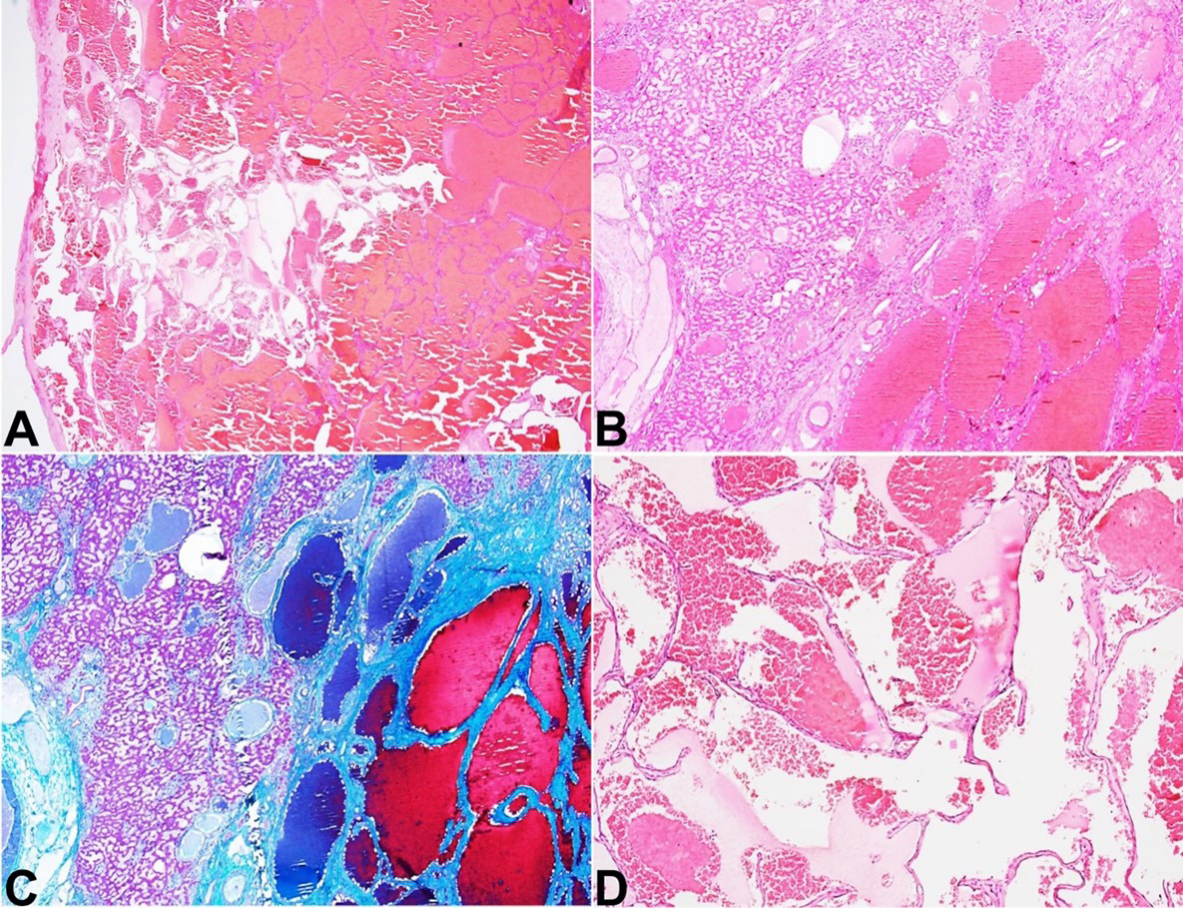
Photomicrographs of the giant cavernous hemangioma. **A –** Scanner view of the largest cavernous hemangioma that was well-encapsulated (H&E; 20x); **B –** Focal insinuation of the margin into the native hepatic parenchyma (H&E; 40x); **C –** highlighted in Masson trichrome stain (C; 40x); **D –** the higher magnification revealing multiple cystically dilated vascular spaces lined by flattened single layer of endothelium containing erythrocytes (H&E; 200x).

Microscopically, all lesions were relatively well-circumscribed (Figure 2A) and partly encapsulated, although there was the microscopic insinuation of the margins into the native hepatic parenchyma (Figure 2B). The irregularity of the margin was highlighted by Masson trichrome stain (Figure 2C). These lesions were vascular and contained numerous, variable-sized, thin-walled, non-anastomosing vascular spaces in a spongiform configuration (Figure 2D). Some of these vascular channels showed irregularly attenuated, thick muscle walls. These vascular spaces were lined by a single layer of flat endothelial cells and contained numerous erythrocytes (Figure 2D). No endothelial multilayering, nuclear atypia, mitosis, or atypical mitosis was evident. Occasional foci of fibrin thrombi were noted within these vascular spaces. The intervening stroma exhibited varying degrees of fibrosis and myxoid degeneration, hyalinization, and calcification. Collection of histiocytes, lymphoplasmacytic infiltrate, lymphoid aggregates, and hemosiderin-laden macrophages were also present in and around the lesions.

**Figure 3:**
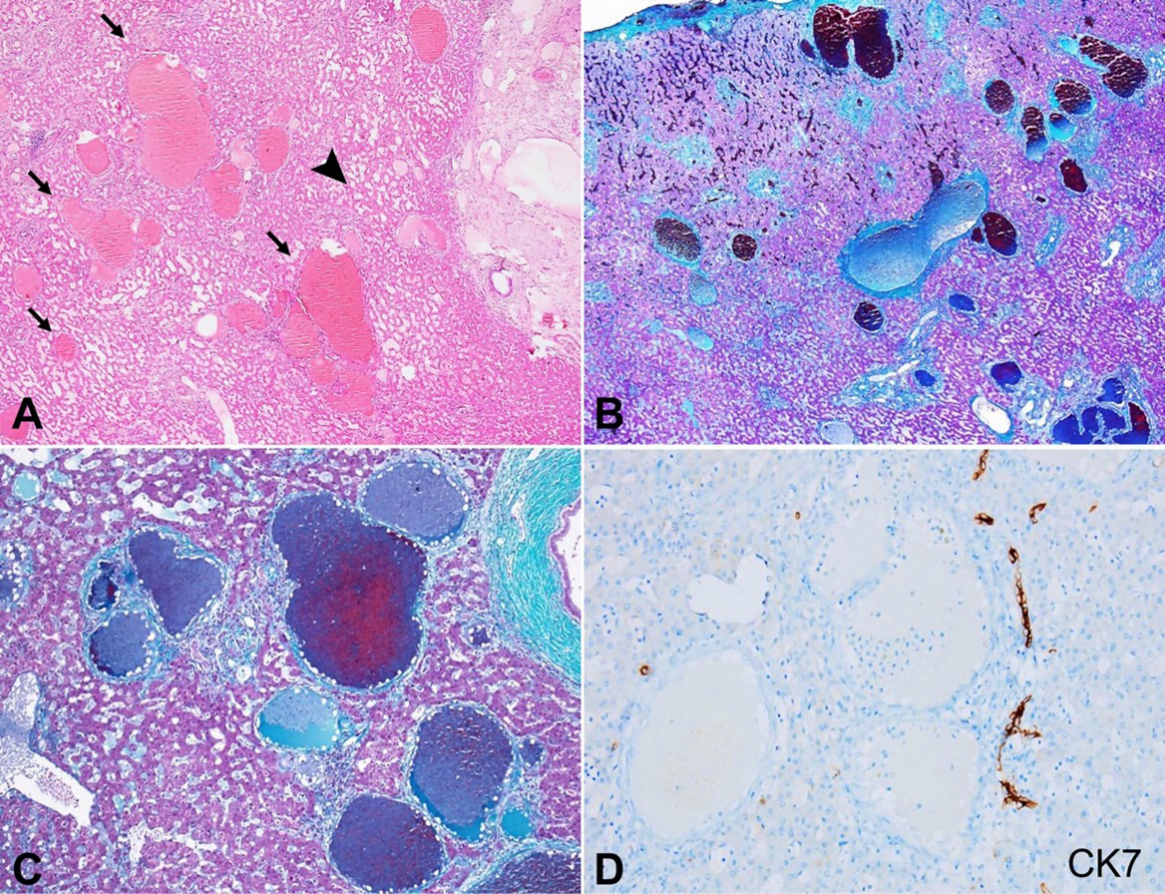
Photomicrographs of the native liver parenchyma. **A –** Variable sized micro-angiomatous lesions characterized by irregular-shaped dilated vascular channels arranged throughout the native liver parenchyma (black arrows) along with sinusoidal dilatation (black arrowhead) (H&E; 20x); **B** and **C –** The locations of the microangiomatous lesions being portal tracts and central veins as highlighted by Masson trichrome stain (B; 20x) (C; 100x); **D –** The retention of the interlobular bile ducts without any ductopenia in CK7 immunostain while the vascular lesions were unstained by CK7 (peroxidase; 200x).

The adjacent liver parenchyma maintained lobular architecture; however, the large majority of the portal tracts and the central veins showed expansion due to the formation of micro-angiomas along with dilatation of the native vessels. These portal tracts and central veins showed irregular shapes due to micro-angioma formation (Figure 3A). These microangiomatous changes were highlighted by Masson trichrome stain (Figures 3B and 3C). Besides, sinusoidal dilatation and congestion, and centrizonal macro-vesicular steatosis (11-33% of the parenchyma) were also evident. CK7 immunostain did not highlight any associated ductopenia (Figure 3D). The surgical margins were involved in these micro-angiomatous lesions. Based on the clinico-radio-histopathological correlation, a possibility of diffuse hepatic hemangiomatosis (DHH) in association with multiple Giant Cavernous Haemangioma (GCH) was suggested.

Postoperatively, the patient was hemodynamically stable and was discharged on day 5 with L-thyroxine (50mcg; O.D.) following endocrinologist consultation. After 6 months of follow-up, she is doing well, and no fresh complaints have been registered. No extrahepatic lesion was noted in further follow-up. The abdominal ultrasonography revealed the presence of 57×47 mm lesion that could not be resected due to its location (close to the middle hepatic vein) along with another small lesion close to the resection limit (segment II) measuring 16×15 mm.

## DISCUSSION

Diffuse hepatic hemagiomatosis (DHH) is an unusual benign vascular lesion of the liver in adults, with less than 20 reported cases in the literature (Table 1). It is characterized by innumerable benign vascular lesions that infiltrate and replace the hepatic parenchyma focally or diffusely.^1^^-^3 The diagnosis of DHH is mostly radiological and/ or histopathological; the latter is required in puzzling cases.^2^ DHH is distinct from cavernous hemangioma, the commonest benign lesion of the liver, by its lack of circumscription and encapsulation, infiltration, and insinuation into the native hepatic parenchyma, and replacement of the native hepatic parenchyma.^3^ Notably, DHH is associated with giant cavernous hemangioma (GCH), a cavernous hemangioma >5 cm in size, in more than half of the cases.^2^^,^^13^^,^^14^ DHH usually affects infants and children. A female preponderance is noted among the adults.^2^ We describe DHH in an adult female of reproductive age group associated with multiple GCHs.

**Table 1:** Detailed summary of clinico-pathological features of reported cases of isolated and systemic adult DHH

Ref	Age/Sex	Clinical features	GCH	Gross	Histopathology	Treatment	Outcome
7	56/F	Generalized bone pain, abdominal pain and abdominal swelling due to hepatic mass	NA	NA	Liver and bone with numerous capillary angiomas consistent with hemangiomatosis. Cystic spaces lined by thin, homogeneously stained endothelial cells. Areas of tumor with numerous intraluminal red blood cells.	Prednisone	Died after 2 weeks
8	30/F	Hepatosplenomegaly, anemia and thrombocytopenia	NA	Liver weighed 6,790 g, was reddish brown, with numerous dark soft areas and whitish discoloration.	Hepatic parenchyma replaced by hemorrhagic cystic tumors. Similar vascular lesion was seen in the spleen, bone marrow, intestine, and peripancreatic lymph nodes.	Radiotherapy Splenectomy	Died 12 months after first presentation due to consumptive coagulopathy
9	22/F	Right upper abdominal pain	NA	Both hepatic lobes with innumerable irregular, sometimes confluent reddish nodules of 0.3-2 cm	Numerous irregularly formed slit-like blood vessels lined by endothelium and slight focal portal fibrosis close to the central vein. The portal triads showed capillary sprouting. The liver capsule was retracted by bands containing capillaries and collagenous fibers.	Stoppage of drug	Regression of lesions
3	35/F	Abdominal pain, weight loss, night sweats and fever	NA	Multiple purple nodules < 2 cm found throughout the tumor; mostly beneath the Glisson’s capsule	Cavernous and diffuse, hemangiomatosis and hemorrhage within the tumor. Innumerable, confluent vascular channels lined by flat endothelial cells linked the hemangiomas.	Left hepatectomy	Recurrence and growth into the right hepatic lobe after two years of surgery. Still progressing at 6 years of follow up.
10	50/F	Tenderness in right upper abdominal quadrant	Yes	Size: 17×14×9 cm, multiple blood-filled honey comb areas of 3 mm to 3 cm	Cavernous hemangioma surrounded by hepatic parenchyma. Vascular channels lined by flattened endothelial cells. No cellular atypia	Right hepatectomy	No mass detected on ultrasonography 9 months post-surgery
11	33/F	Abdominal distension, edema and hepatomegaly	NA	NA	Prominent cavernous vascular proliferation and fibrosis without angiosarcomatous components	None	Patient expired due to liver failure 10 days after admission
12	33/F	Abdominal distension and shortness of breath	NA	NA	Variably dilated non-anastomotic vascular spaces lined by flat endothelial cells (CD 34 +).	NA	Died of liver failure after 12 days while waiting for liver transplant
13	78/M	Abdominal pain and distension	Yes	NA	Cavernous hemangioma with irregularly dilated non-anastamotic vascular spaces lined by flat endothelial cells alternating with normal hepatic parenchyma	None	Improvement of discomfort and quality of life after 9 months follow up.
14	35/F	Epigastric pain and abdominal fullness	Yes	The resected tumor was 20× 14× 8.5 cm in size and 910 g in weight	Hemangiomatous lesions were scattered around the Glisson’s capsule	Right hepatectomy	Discharged on POD9 without identifiable lesions
15	68/M	Asymptomatic	NA	NA	Endothelial-lined sinusoidal proliferation with erythrocyte content, consistent with hepatic hemangiomatosis	None	Stable at two years of follow up
16	59/M	Asymptomatic	NA	NA	H&E showed hemangiomas with (CD34+, CD31+, anti-desmin negative) no cellular atypia.	NA	Died due to hepatic failure
17	50/F	Abdominal pain, hepatomegaly and dyspnea	Yes	Well-defined sponge-like reddish brown mass. Remaining parenchyma with similar small lesion.	Main mass with variable-sized vascular spaces, lined by endothelial cells. Multiple scattered small hemangiomas also present.	Liver transplant	Stable for 1.5 years after surgery
18	83/F	Abdominal distension and hepatomegaly	NA	Multiple characteristic dark red nodules on liver surface (laparoscopic finding)	Irregularly dilated vascular spaces, mostly close to the portal tract, lined by endothelial cells (CD34+ and CD31+) without cellular atypia	Bevacizumab	Died 12 months after diagnosis due to multiple organ failure (KMS, hemolytic anemia, heart failure, DIC)
19	62/M	Asymptomatic	NA	NA	Focal areas of sinusoidal dilatations lined by flattened endothelial cells.	NA	NA
20	29/F	H/O Endometriosis and received OCPs	Yes	Lesion in segment IVa with surrounding changes and a well-defined lesion containing 6.5-cm blood clot in segment IVb	Disseminated aggregate of blood vessels lined by endothelium without atypia.	Stoppage of drug and left liver lobectomy	No recurrent liver lesion after 1 year of surgery.
21	40/F	Abdominal pain and distension	Yes	NA	DHH with a giant hemangioma. No mitosis. STAT6, WT1, Desmin, p53, D2-40 were negative.	Chemotherapy, trans-arterial embolization and Liver transplant	Good condition after 6 months of follow-up.
4	63/M	Abdominal bloating and constipation	Yes	NA	NA	None	NA
6	62/M	Backache, Hepatomegaly	NA	NA	Lesion filled with red blood cells, lined by flat endothelial cells (CD34+) without atypia.	None	Good condition after 6 months of follow-up.
22	56/F	Chronic abdominal discomfort	Yes	NA	NA	NA	NA
Our case	37/F	Right upper quadrant fullness and shortness of breath	Yes	Well encapsulated, dark red to tan-brown colored masses and few perilesional cherry-colored honeycomb foci.	Microscopic insinuation of the vascular lesion margins into the normal hepatic sinusoids. Lesions with variably-sized to large, thin-walled, non-anastomosing vascular spaces, and irregularly attenuated thick muscle walls. Vascular spaces lined by single layer of endothelial cells. Fibrin thrombi were noted within these vascular spaces. Stroma with fibrosis, myxoid degeneration, hyalinization, and calcification.	Surgical enucleation	Good condition after 6 months of follow-up.

M = male; F = female; GCH = giant cavernous hemangioma; OCPs = oral contraceptive pills; NA = not available; KMS = Kasabach–Merritt syndrome; DIC = disseminated intravascular coagulation; H/O = history of.

DHH usually presents with abdominal pain and distension similar to the index case. The presence of jaundice can result from the parenchymal replacement by the vascular lesions, mass effect, and hepatic dysfunction due to ischemia. The mass effect with an obstructive component appears significant due to the presence of jaundice along with clay-colored stools in the absence of any ductopenia. A normal to mild elevation of the liver function tests has been documented in the literature.^2^^,^^3^ The association of adult DHH with oral contraceptive (OC) and metoclopramide has been reported.^20^^,^^23^ We could not document any history of OC or metoclopramide intake in our case. The association of DHH and hypothyroidism is known in infants, although no such association is documented in adult DHH. In infants, DHH is associated with a challenging form of consumptive hypothyroidism which requires prompt treatment with propranolol and an increased dose of L-thyroxine to prevent long-term sequelae.^24^ The association of adult DHH and hypothyroidism in the index case could be fortuitous, although further study is required before concluding.

On CT scan, DHH is observed as a nodular or infiltrative hypoattenuating lesion with indistinct borders in contrast to GCH. This feature was missed on CECT, although appreciated retrospectively in the index case. Delayed centripetal enhancement is the characteristic finding in contrasted studies, however the infiltrative borders can be overlooked in the case of isolated DHH without multisystemic involvement, similar to the index case.^10^^,^^14^ Magnetic resonance imaging (MRI) is the most sensitive technique and shows lesions with hyperintensity on T2 weighted sequencing with progressive contrast enhancement.^4^^,^^6^ The definitive diagnosis requires histopathological examination in doubtful and complicated cases. DHH can occur either in separate lobes or simultaneously in both hepatic lobes. Some patients have extrahepatic lesions in other organs like the spleen, intestine, lungs, skin, nervous system, adrenal, and bone marrow.^7^^,^^8^ The radiology is particularly important and inevitable for assessing the extrahepatic involvement and follow-up of DHH.

Histological features of DHH include thin-walled, cystically dilated, non-anastomosing, vascular channels with infiltration of the native hepatic parenchyma and without any encapsulation/ circumscription, the latter two features separating it from a CH. These vascular channels are lined by a single layer of flat endothelial cells with no significant nuclear atypia. Secondary changes like degeneration of the vessel wall, calcification, intracavitary erythrocyte deposition, and thrombosis are usually seen similar to CH.^1^^,^^3^^,^^4^ Kasabach–Merritt syndrome and disseminated intravascular coagulation are commonly reported complications of DHH.^8^^,^^25^ The index case did not show any feature of consumption coagulopathy or any extrahepatic involvement. The clinical and histological differential diagnoses of hypervascular hepatocellular carcinoma, polycystic liver disease, mesenchymal hamartoma, inflammatory hepatocellular adenoma, haemangioendothelioma, and angiosarcoma could not be entertained in the index case devoid of any typical histopathology.^5^^,^^26^

The occurrence of adult DHH is an uncommon event although its localized form, namely CH is the commonest benign hepatocytic lesion. Table 1 highlights the clinical and histopathological features of many previously reported cases of adult DHH (N=19). Among them, 16 cases presented isolated DHH, while two cases showed the systemic occurrence of hemangiomatosis. The presence and absence of GCH were noted in nine cases and two cases, respectively, including ours, while nine cases did not report the association with GCH. Treatment of DHH is varied, and surgical resection can be performed if the tumor margin is clear and confined to one lobe.^10^^,^^22^^,^^26^ However, the surgical resection is often unsatisfactory in the deep-seated lesions, and in lesions close to major vessels, similar to the index case. Radiation and anti-VEGF therapy can be tried before liver transplantation in inoperable cases.^17^^,^^18^^,^^21^ Besides, cessation of drugs like OC/ metoclopramide may be of help.^9^^,^^20^ The prognosis of DHH is uncertain due to its rarity. Liver failure is the most common complication followed by Kasabach-Merritt syndrome, disseminated intravascular coagulation (DIC), and heart or multiple organ dysfunction.^7^^,^^8^^,^^11^^,^^12^^,^^18^ There was death of six adult patients and recurrence of the lesion in one patient following the diagnosis of DHH, while follow-up was not reported in three cases (Table 1). Recurrences are common and usually related to incomplete excision as well as recruitment of collateral arterial flow into a low resistance vascular bed.^3^ The current case was surgically managed with follow-up of the deep-seated in-situ lesions of the patient.

## CONCLUSION

The index case illustrates an isolated adult DHH in association with GCH. The diagnosis can be suspected using radiological modalities like USG, CT, or MRI; however, a definitive diagnosis requires histopathological evaluation. The characteristic peripheral or delayed enhancement on CT coupled with the morphology on which a diffuse replacement and lack of capsular restriction of these vascular lesions are usually helpful. Surgical resection alone might not be satisfactory if the involvement of bilateral lobes and a deep-seated location is encountered wherein radiation can be tried. Recurrences are common, and liver transplantation is the last resort to treatment.
